# Addressing health disparities using multiply imputed injury surveillance data

**DOI:** 10.1186/s12939-023-01940-4

**Published:** 2023-07-03

**Authors:** Yang Liu, Amy F. Wolkin, Marcie-jo Kresnow, Thomas Schroeder

**Affiliations:** 1 Division of Injury Prevention, National Center for Injury Prevention and Control, U.S. Centers for Disease Control and Prevention, Atlanta, GA USA; 2 Division of Hazard and Injury Data Systems, U.S. Consumer Product Safety Commission, Bethesda, MD USA

**Keywords:** Health Disparity, Missing Data, Multiple Imputation, Non-fatal Assault Injury, National Electronic Injury Surveillance System-All Injury Program (NEISS-AIP)

## Abstract

**Background:**

Assessing disparities in injury is crucial for injury prevention and for evaluating injury prevention strategies, but efforts have been hampered by missing data. This study aimed to show the utility and reliability of the injury surveillance system as a trustworthy resource for examining disparities by generating multiple imputed companion datasets.

**Methods:**

We employed data from the National Electronic Injury Surveillance System-All Injury Program (NEISS-AIP) for the period 2014–2018. A comprehensive simulation study was conducted to identify the appropriate strategy for addressing missing data limitations in NEISS-AIP. To evaluate the imputation performance more quantitatively, a new method based on Brier Skill Score (BSS) was developed to assess the accuracy of predictions by different approaches. We selected the multiple imputations by fully conditional specification (FCS MI) to generate the imputed companion data to NEISS-AIP 2014–2018. We further assessed health disparities systematically in nonfatal assault injuries treated in U.S. hospital emergency departments (EDs) by race and ethnicity, location of injury and sex.

**Results:**

We found for the first time that significantly higher age-adjusted nonfatal assault injury rates for ED visits per 100,000 population occurred among non-Hispanic Black persons (1306.8, 95% Confidence Interval [CI]: 660.1 – 1953.5), in public settings (286.3, 95% CI: 183.2 – 389.4) and for males (603.5, 95% CI: 409.4 – 797.5). We also observed similar trends in age-adjusted rates (AARs) by different subgroups for non-Hispanic Black persons, injuries occurring in public settings, and for males: AARs of nonfatal assault injury increased significantly from 2014 through 2017, then declined significantly in 2018.

**Conclusions:**

Nonfatal assault injury imposes significant health care costs and productivity losses for millions of people each year. This study is the first to specifically look at health disparities in nonfatal assault injuries using multiply imputed companion data. Understanding how disparities differ by various groups may lead to the development of more effective initiatives to prevent such injury.

**Supplementary Information:**

The online version contains supplementary material available at 10.1186/s12939-023-01940-4.

## Introduction

Over a million people are treated in emergency departments for nonfatal assault injuries in the United States every year [1]. Such injuries are also a leading cause of mortality among children and young adults, and many result in life-long disabilities and health consequences [2]. These health issues can impose heavy costs on individuals and society. The Centers for Disease Control and Prevention estimates the 2019 nonfatal injury costs $2.0 trillion in medical care and lost productivity, which is more than four times as high as the 2013 estimate ($457 billion) [3]. According to Moore et. al., there are disparities in injury incidence and the burden of injury falls disproportionately on “communities of color, those who are economically disadvantaged, and those who are geographically isolated” [4]. Understanding how health disparities differ by various groups may lead to the development of more effective initiatives to prevent such injury. Investigating disparities in health between groups requires accurate identification and categorization [5, 6]. However, information about key indicators for identification such as race/ethnicity, sex, age, and geographic location may be missing or not collected in large survey data [7–9]. This lack of complete information may cause inaccurate estimation of disparities, especially when the proportion of missing data is high. Efforts to address health disparities in injury research have been hampered by missing data [10–12]. Complete data are essential for identifying disparities, minimizing bias, and for improving statistical power and efficiency.

NEISS-AIP collects data from a nationally representative sample of hospital emergency departments (EDs) using specific guidelines for recording the primary diagnosis and mechanism of all types of injuries treated [1, 13]. It can be used to (1) measure the magnitude and distribution of nonfatal injuries in the United States; (2) monitor unintentional and violence-related injuries over time; (3) discover emerging injury problems; and (4) set national priorities. Analysing and disseminating these surveillance data will help support the mission of reducing all types and causes of injuries in the United States [14]. However, as with any large-scale data collection effort, NEISS-AIP often contains missing data. For the data years of 2014–2018, more than half (57.1%) of records reported at least one missing variable. In particular, patient race/ethnicity (RACE) and location of injury (LOC) had the highest proportions of missing data (32.7% for RACE and 35.3% for LOC). Identifying disparities requires accurate data on health status and individual determinants of health for subgroups of the population. These missing key indicators can hinder the use of NEISS-AIP in investigating health disparities.

Missing data continues to limit the analysis of health-related disparities and their causes. The most widely adopted strategy to overcome the missing data barrier is to omit observations with missing values and perform a complete case analysis (CCA). However, the cumulative effect of missing data in several variables often leads to exclusion of a substantial proportion of original sample. The results from CCA may be biased because the complete case can be unrepresentative of the full population. CCA may suffer from the loss of statistical precision and risk of bias, since incorrect handling of missing data might result in drawing the wrong conclusion, as effect estimates and error measurements may be altered [15]. Multiple imputation (MI) is widely recognized as another standard approach for handling missing data, particularly when data are partially missing for multiple variables [16–18]. The missing values are imputed based on a model that relates the missing variable to observed variables, which generates multiple complete datasets without missingness. Estimates and standard errors (SE) are calculated for each imputation set and pooled into one overall estimate and SE. MI predicts data based on the known variables with the incorporation of missing data uncertainty, which leads to more accurate estimates than single imputation. It is a powerful and statistically valid method for creating imputations in large datasets with complex data structures [19, 20].

Accounting for missing data is essential for facilitating injury research into health disparities [21–23]. The present study aims to make NEISS-AIP a more useful and reliable resource for examining disparities by generating multiple imputed companion datasets. We conducted a comprehensive simulation study to identify the appropriate MI method for handling missing data in NEISS-AIP. We selected the multiple imputations by fully conditional specification (FCS MI) to generate the imputed companion data to NEISS-AIP 2014–2018. These complete data were used to further assess health disparities by race and ethnicity, location of injury, and sex in nonfatal assault injuries treated in U.S. hospital EDs. To our knowledge, this study is the first to assess health disparities in injury using multiple imputed NEISS-AIP data.

## Methods

### NEISS-AIP 2014–2018 Data

The NEISS-AIP is designed to provide national incidence estimates of all types and external causes of nonfatal injuries and poisonings treated in U.S. hospital EDs [13]. Data on injury-related visits were obtained from a national sample of 66 of 100 NEISS hospitals, which were selected as a stratified probability sample of hospitals in the United States with a minimum of six beds and a 24-h ED. Data were weighted by the inverse of the probability of selection to produce national estimates. The sample included separate strata for very large, large, medium and small hospitals, defined by the number of annual ED visits per hospital. Trained, onsite hospital coders abstracted data for injury-related cases from ED records at NEISS hospitals. NEISS-AIP is providing data on approximately 600,000 cases annually. Data collected include age, race/ethnicity, gender, principal diagnosis, primary body part affected, consumer products involved, disposition at ED discharge, the locale where the injury occurred, work-relatedness, and a narrative description of the injury circumstances. Also, major categories of external cause of injury and of the intent of injury are being coded for each case in a manner consistent with the International Classification of Diseases, Tenth Revision, Clinical Modification (ICD-10-CM) coding rules and guidelines. NEISS-AIP provides an excellent data source for monitoring national estimates of injuries over time. However, incomplete NEISS-AIP data poses challenges for identifying health disparities and for analysing the underlying causes.

### Multiple Imputation (MI) Method

To create a full dataset and to minimize bias due to systematic differences between complete records and those with missing data, MI was performed. MI is a three-step approach following Rubin’s rules [24] to estimation of incomplete data: (1) imputation of missing values from a so-called “imputation model” repeated *m* times, which results in the m complete imputed data set; (2) the fitting of an “analysis model” (i.e., the model of interest) to each of the *m* imputed data sets separately; (3) pooling of the *m* sets of estimates thus obtained to give an overall set of estimates and corresponding standard errors [25–31]. The general procedure for MI was described in Supplementary Text A. To identify important covariates in the imputation model, Cramer’s V statistic was used to measure the correlation between the missing variable and covariates [30]. The covariate was included in the imputation model if it correlated with an absolute value of a Cramer’s V greater than 0.05 (all covariates used are summarized in Supplementary Table S1). All statistical analyses, including MI methods, were conducted using SAS version 9.4 (SAS Institute, Cary, NC).

Two major approaches in MI exist: joint modeling (JM) and fully conditional specification (FCS) [27]. JM imputations involve specifying a multivariate distribution for the missing data and drawing an imputation from their conditional distributions using Markov Chain Monte Carlo (MCMC) techniques. FCS imputations are generated sequentially variable-by-variable by specifying an imputation model for each missing variable given the other variables. FCS MI is a more flexible approach for creating imputations in large datasets which include both categorical and continuous variables [28, 29].

### Simulation study

A comprehensive simulation study was performed on the 2018 NEISS-AIP data year to illustrate and to distinguish the performance of various missing data approaches. The missing data pattern can influence the amount of information transferred between variables, so we first investigated the missing pattern in the NEISS-AIP 2018 data to determine all possible intersections between different missing variable sets (Supplementary Table S2, total fifty-four patterns with at least one missing variable were obtained). We then developed a Venn diagram to visualize the intersections and the cumulative missing percentage.

Given the complex data structure and the large sample size (over 600,000 cases collected in NEISS-AIP annually), it is impossible to simulate the NEISS-AIP using classic simulations. We developed a new simulation dataset by imposing the missing data patterns on the subset of fully observed data in NEISS-AIP 2018. The missingness in the simulated data was generated using random sampling without replacement to mimic all possible intersections among missing variables. Each observation in the data set had an equal chance of being selected, once selected it couldn’t be chosen again. SAS PROC SURVEYSELECT procedure was applied to generate a variety of random samples for the missing pattens [32]. As the true values of these missing data were already known from the non-missing data, the accuracy of imputed values could be assessed using the non-missing data as the standard control.

To evaluate the imputation performance more quantitatively, we developed a new method based on the Brier Skill Score (*BSS*) to observe the performance difference [33–35]. The detailed algorithm of the *BSS* based new method was described in the Appendix**. ***BSS* is useful for envisioning the difference in imputation performance and for measuring the accuracy improvements of probabilistic predictions. We performed the *BSS* comparison of the imputation performance for all categorical missing variables by two MI methods (JM and FCS), in which CCA was chosen as the reference strategy.

As the key covariate of interest, race/ethnicity information is vital for measuring disparities across groups [8]. To assess the impact of MI on the overall distribution, we determined the distributions of race/ethnicity among the simulation dataset (before imputation), JM and FCS imputed data (after imputation), and the standard control (the non-missing data). For our analysis, race/ethnicity was recoded into four categories to make them compatible with available annual bridged-race population estimates used as denominators for the injury rates: White, non-Hispanic (White NH); Black or African American, non-Hispanic (Black NH); Hispanic; and Other NH. We combined Asian, non-Hispanic (Asian NH); American Indian or Alaska Native, non-Hispanic (AIAN NH); and Pacific Islander, non-Hispanic (PI NH) into one group as Other NH. Race bridging refers to making data collected using one set of race categories consistent with data collected using a different set of race categories, to permit estimation and comparison of race-specific statistics at a point in time or over time. Prior to 2021, reporting injury rates in these four mutually exclusive categories is consistent with mortality reporting from National Center of Health Statistics and incidence reporting from National Cancer Institute [36, 37].

We compared crude nonfatal assault injury rates within the White NH group to illustrate the bias in injury rate estimates when using various missing data approaches. White NH group was selected for illustrative purposes since it had the largest proportion of population in the data. Nonfatal assault injuries were limited to those injuries treated in the ED and resulting from physical violence by one or more persons; sexual assaults and injuries from legal intervention were excluded [13]. The absolute deviations of nonfatal assault injury rate estimates were calculated using the standard control rate as the reference. The 2014–2018 U.S. Census Bureau bridged-race population estimates were used to calculate nonfatal assault injury rates per 100,000 population.

### Assessing disparities using FCS imputed companion data

FCS MI showed the best overall imputation performance based on the simulation study. We then implemented this approach on the NEISS-AIP data to impute the missing data in each year from 2014 to 2018 (The general SAS coding procedure for PROC MI using FCS statement was included in Supplementary Text B). Finally, all years of imputed data were merged to generate an imputed companion dataset to the NEISS-AIP 2014–2018 data.

Next, we assessed health disparities by race/ethnicity, location of injury, and sex using imputed companion data. To allow for accurate comparisons between groups with different age distributions, we calculated age-adjusted average annual rates of nonfatal assault injury per 100,000 population among hospital ED visits by RACE, LOC, and SEX. The estimated nonfatal assault injury rates were age-adjusted to the 2000 U.S. standard population. We also displayed trend analysis for age-adjusted nonfatal assault injury rates by different groups in each year from 2014 to 2018. The significant differences in nonfatal assault injury rates across various groups were tested using t-tests, where p-values < 0.05 were considered statistically significant. Analyses were conducted using SAS 9.4 (SAS Institute, Inc, Cary, NC), and 95% CIs and statistical tests accounted for the sampling weights and complex survey design. Data were weighted by the inverse of the probability of selection to provide national estimates.

## Results

### Missing data in NEISS-AIP 2014–2018

**Fig. 1:**
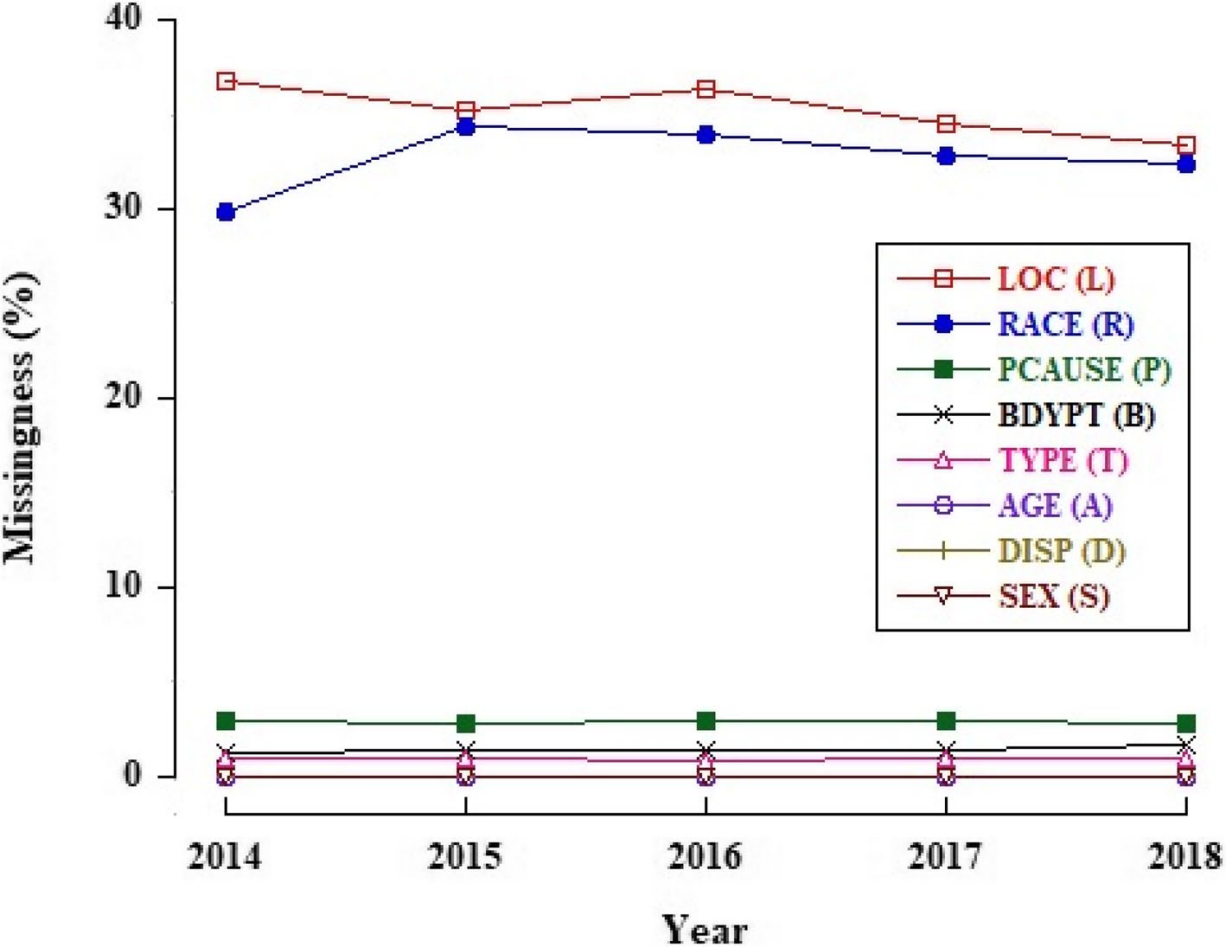
Trends in missing proportions for variables with missingness^a^ in NEISS-AIP^b^ data from 2014 to 2018. Patient race/ethnicity (RACE) and location of injury (LOC) show the highest proportions of missing data across years (> 30%). ^a^LOC: location where the injury occurred. RACE: race and ethnicity of patient. CAUSE: external cause of injury. BDYPT: primary body part affected. TYPE: work-relatedness. AGE: patient age in year. DISP: disposition at emergency department discharge. SEX: gender of patient. ^b^NEISS-AIP: National Electronic Injury Surveillance System-All Injury Program

Table 1 shows the unweighted counts and percentages for all missing variables in NEISS-AIP data from 2014–2018. Due to the cumulative effect of missing data, more than half (57.1%) of records were missing at least one variable and only 42.9% had fully complete (non-missing) data. Figure 1 displays the trend for all missing variables from 2014 to 2018. RACE and LOC had the highest proportions of missing data across years (> 30%).

**Table 1:** Frequency analysis of missingness: unweighted counts and percentages for missing variables in NEISS-AIP^a^, United States, 2014–2018

Missing Variables^b^	2014		2015		2016		2017		2018		2014–2018	
	**n**	**%**	**n**	**%**	**n**	**%**	**n**	**%**	**n**	**%**	**N**	**%**
**LOC**	227,655	36.8	215,807	35.2	226,014	36.4	220,506	34.5	193,958	33.4	**1,083,940**	**35.3**
**RACE**	184,732	29.8	210,518	34.4	211,600	34.0	209,781	32.8	188,437	32.4	**1,005,068**	**32.7**
**CAUSE**	17,924	2.9	16,897	2.8	18,557	3.0	19,031	3.0	16,048	2.8	**88,457**	**2.9**
**BDYPT**	7,386	1.2	8,306	1.4	8,876	1.4	8,691	1.4	9,862	1.7	**43,121**	**1.4**
**TYPE**	5,507	0.9	5,359	0.9	4,861	0.8	5,468	0.9	5,450	0.9	**26,645**	**0.9**
**AGE**	199	0.03	254	0.04	251	0.04	238	0.04	198	0.03	**1,140**	**0.04**
**DISP**	16	0.003	6	0.001	7	0.001	204	0.03	15	0.003	**248**	**0.008**
**SEX**	8	0.001	13	0.002	7	0.001	22	0.003	23	0.004	**73**	**0.002**
**Missing Total** ^**c**^	346,357	55.9	349,875	57.1	366,692	59.0	368,159	57.6	324,396	55.8	**1,755,479**	**57.1**
**Non-missing Total** ^**d**^	272,986	44.1	262,545	42.9	254,880	41.0	271,355	42.4	256,529	44.2	**1,318,295**	**42.9**
**Total Observations**	619,343		612,420		621,572		639,514		580,925		**3,073,774**	

^a^ *NEISS-AIP* National Electronic Injury Surveillance System-All Injury Program

^b^ *LOC* location where the injury occurred, *RACE* race and ethnicity, *CAUSE* external cause of injury, *BDYPT* primary body part affected, *TYPE* work-relatedness, *AGE* age in years, *DISP* disposition at emergency department discharge, *SEX* gender

^c ^Missing Total: the cumulative missing where observations had at least one missing variable

^d ^Non-missing Total: observations had no missing values

### Simulation study

**Fig. 2:**
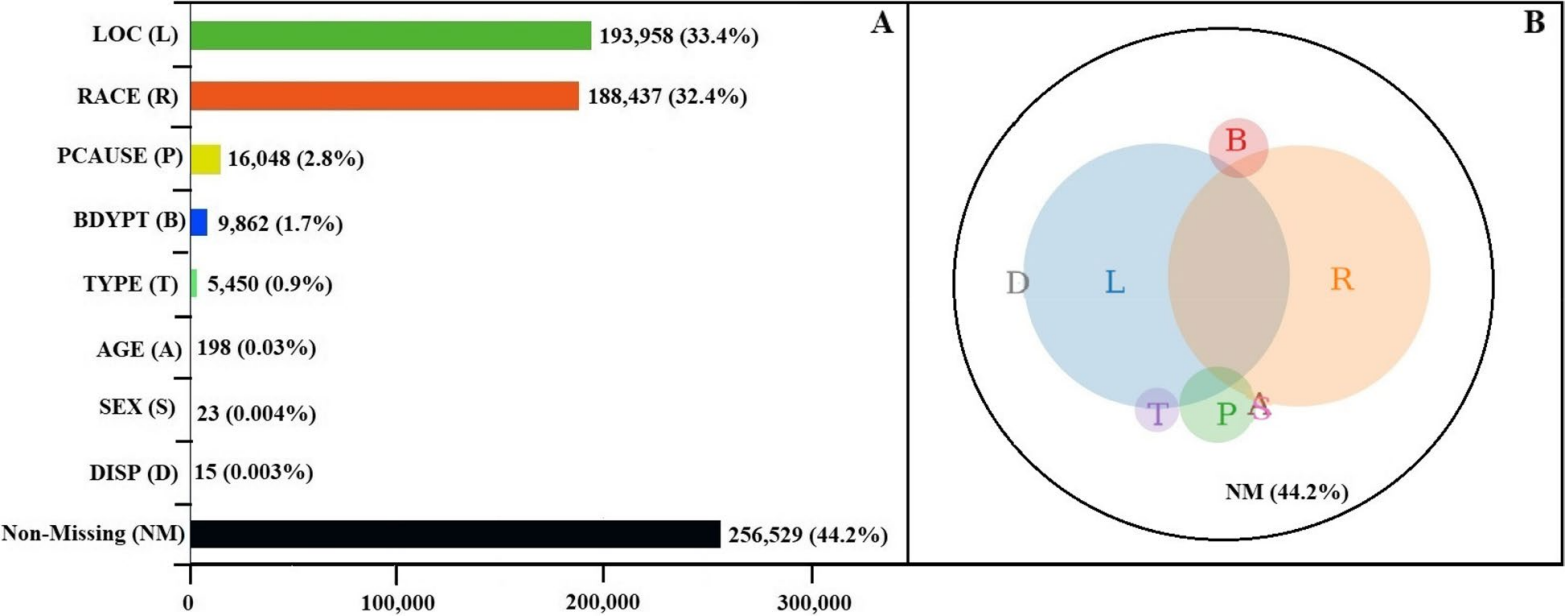
Analyzing missing data patterns of NEISS-AIP^a^ 2018 data: A. Bar chart of unweighted counts and proportions for both missing and non-missing data^b^; B. The Venn diagram for presenting the missing data patterns^b^. Note: A (Age) and S (Sex) overlay in Fig. 2B and represent small population sizes. A and S both intersect with P (CAUSE) and R (RACE). D (DISP) represents a small population size, and it intersects with L (LOC) only.^a^NEISS-AIP: National Electronic Injury Surveillance Systems-All Injury Program. ^b^LOC (L): location where the injury occurred. RACE (R): race and ethnicity of patient. CAUSE (P): external cause of injury. BDYPT (**B**): primary body part affected. TYPE (T): work-relatedness. AGE (**A**): patient age in year. DISP (D): disposition at emergency department discharge. SEX (S): gender of patient

Figure 2 displays the proportions of missingness of data and data patterns in NEISS-AIP for 2018. More than 30% of observations in hospital ED visits were missing information on LOC (33.4%) and RACE (32.4%). Missing data for other key variables ranged from 2.8% to less than 1% (Fig. 2A). In addition, a complex set of overlapping missing data patterns exists among these missing variables (Supplementary Table S2). A Venn diagram (Fig. 2B) was developed to visualize all possible logical relationships among a finite collection of the different missing sets.

**Fig. 3:**
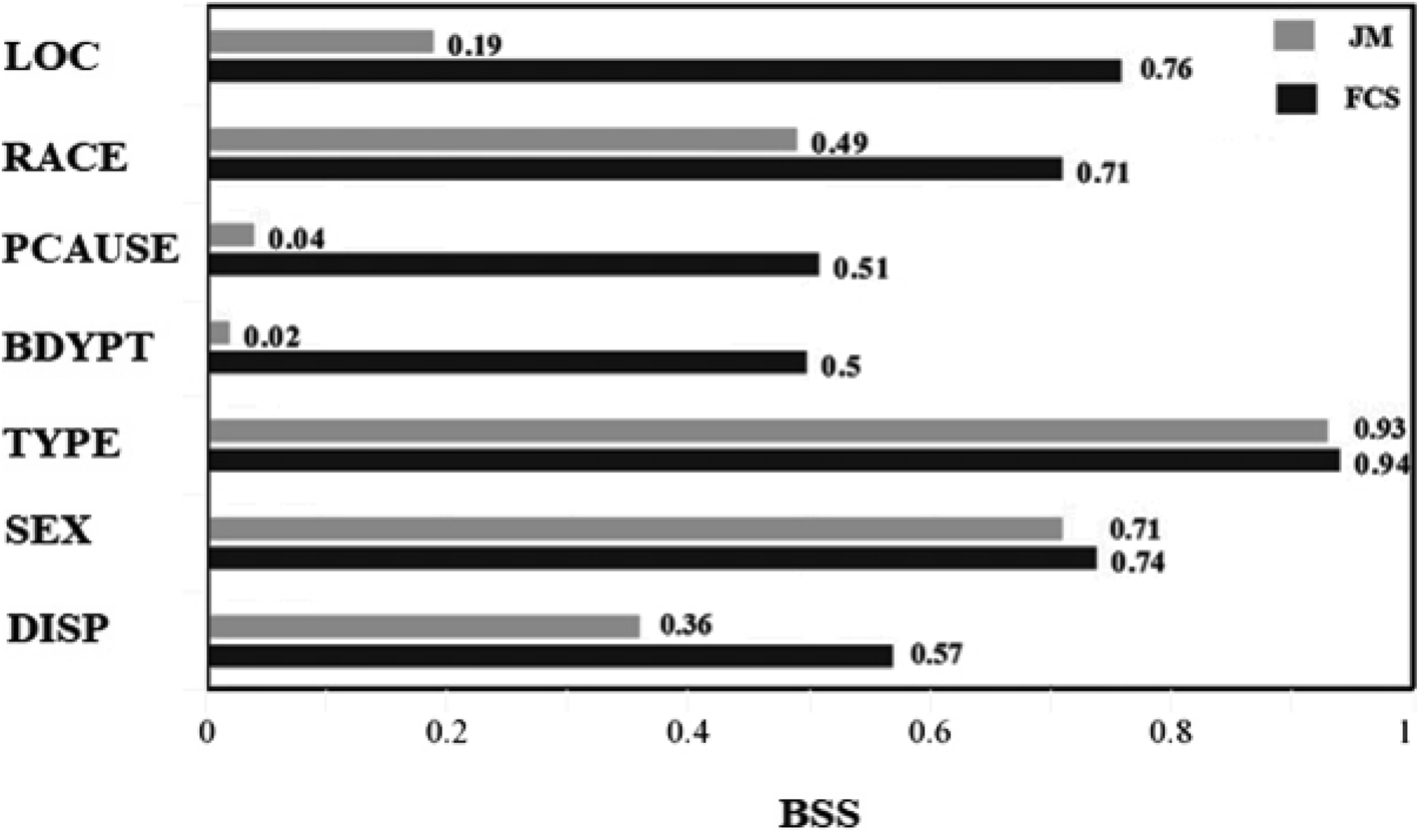
Brier Skill Score (*BSS*)^a^ comparison for evaluating imputation performance on simulation data^b^ by using the different models (JM and FCS). ^a^ *BSS* indicates the degree of skill improvement. A *BSS* range from 0 to 1: 0 means no improvement in accuracy and 1 means a perfect accuracy of prediction. ^b^Simulation data was developed by imposing the missing data patterns on the subset of fully observed data in National Electronic Injury Surveillance System-All Injury Program (NEISS-AIP) 2018. JM: joint modelling. FCS: fully conditional specification. LOC: location where the injury occurred. RACE: race and ethnicity of patient. CAUSE: external cause of injury. BDYPT: primary body part affected. TYPE: work-relatedness. AGE: patient age in year. DISP: disposition at emergency department discharge. SEX: gender of patient

Figure 3 displays the *BSS* comparison for all the missing categorical variables to visualize the imputation performance difference using two MI methods (JM and FCS). The FCS method far exceeded the JM method in accurately imputing missing data in most instances except for TYPE and SEX, where the two performed almost identically. Overall FCS shows larger *BSS* than JM, implying that FCS is associated with more accurate predicted probabilities than JM.

To assess the impact of MI on the overall distribution of race/ethnicity, we determined the distributions before imputation (simulation data) and after imputation (JM or FCS imputed data) and compared them with the true estimates in the standard control data (Figure S1, Supplementary). Before imputation, the race/ethnicity distribution is significantly different from the true proportions of standard control. After imputation, JM tends to underestimate the true proportion for White NH and Other NH, and to overestimate the true proportion for Black NH and Hispanics. Unlike JM, FCS shows the minimal effect on the true overall distribution of race/ethnicity after imputation. Adding the FCS imputed cases of race/ethnicity to the data has little impact on the known overall distribution of the respondents by race/ethnicity.

Table 2 shows the comparison of estimated crude rates of nonfatal assault injuries for the White NH group under different approaches for addressing missing racial/ethnic data. Dropping persons with missing race/ethnicity (using CCA) not only causes loss in statistical power but also results in significant bias in rate estimation compared to the standard control rate (32.3% absolute deviation from the true rate). After imputation, the absolute deviations are calculated as 14.1% for JM imputation and only 0.3% for FCS imputation. This simulation provides strong evidence that FCS MI generally produces unbiased estimates when compared with the standard control. FCS MI shows the best overall results for addressing high proportions of missingness in NEISS-AIP data.

**Table 2:** Comparison of the different missing data strategies in the simulation study: estimating** c**rude rate of nonfatal assault injuries treated in EDs (Emergency Departments) per 100,000 population among non-Hispanic white individuals

	**Weighted Number of Injuries**	**Crude Rate per 100,000 Population** ^**b**^	**95% CI**	**Absolute Deviation** ^**c**^ $|\frac{\left({\varvec{R}}{\varvec{a}}{\varvec{t}}{\varvec{e}}-{\varvec{S}}{\varvec{T}}{\varvec{D}}\right)}{{\varvec{S}}{\varvec{T}}{\varvec{D}}}|$
CCA	217,080	244.5	(175.3, 313.7)	32.3%
JM MI	275,609	310.4	(223.3, 397.5)	14.1%
FCS MI	321,759	362.4	(255.0, 469.8)	0.3%
Standard Control^a^	320,629	361.2	(257.9, 464.5)	-

The simulation study built on real non-missing data from National Electronic Injury Surveillance System-All Injury Program (NEISS-AIP) 2018 was conducted to identify the appropriate MI model for handling missing data

*Abbreviations*: *NH* non-Hispanic, *CCA* complete case analysis, *JM* joint modeling, *MI* multiple imputation, *FCS* fully conditional specification, *EDs* Emergency Departments

^a ^Non-missing data was used as standard control data

^b^ Excludes sexual assault cases

^c ^Absolute deviation was calculated by using the standard control rate as the reference

### Assessing disparities using FCS imputed companion data

Table 3 displays the estimated age-adjusted average annual rate of nonfatal assault ED visits per 100,000 population by RACE, LOC, and SEX. For the 2014–2018 study period, Black NH persons showed a significantly higher nonfatal assault injury rate per 100,000 population (1306.8) compared to their counterparts (347.8 for White NH persons, 366.0 for Hispanic persons, and 291.2 for Other NH persons). The rate of nonfatal assault injuries was significantly higher in a public setting than at home (286.3 vs. 200.4). Males also had a significantly higher nonfatal assault injury rate compared to females (603.5 vs. 369.5).

**Table 3:** Assessing health disparities using Fully Conditional Specification Imputed Companion Data, NEISS-AIP^a^, 2014–2018: Estimating Age-adjusted^b^ Average Annual Rate of Nonfatal Assault Injury per 100,000 Population for United States Hospital Emergency Department Visits, by Race/Ethnicity, Location of Injury and Sex

Characteristic	Categories	Unweighted Number of Injuries^d^	National Estimate (%)	Age-adjusted Average Annual Rate (95% CI)	T-tests
**Total**	___	142,876	7,610,896 (100)	486.7 (352.4, 621.1)	___
**Race and Ethnicity**	Black NH^c^	66,765	3,048,912 (40.1)	1306.8 (660.1, 1953.5)	Ref
	White NH	43,694	3,178,305 (41.8)	347.8 (261.4, 434.1)	p = 0.001
	Hispanic	24,851	1,046,120 (13.7)	366.0 (136.4, 595.5)	p = 0.001
	Other NH	7,566	337,560 (4.4)	291.2 (125.1, 457.3)	p = 0.001
**Location of Injury**	Public Setting	85,115	4,479,969 (58.9)	286.3 (183.2, 389.4)	Ref
	Home	57,761	3,130,927 (41.1)	200.4 (162.5, 238.3)	p = 0.003
**Sex**	Male	90,683	4,754,049 (62.5)	603.5 ( 409.4, 797.5)	Ref
	Female	52,193	2,856,846 (37.5)	369.5 (288.7, 450.4)	p = 0.002

^a ^NEISS-AIP: National Electronic Injury Surveillance System-All Injury Program

^b ^Age-adjusted to the 2000 U.S. standard population

^c ^NH: Non-Hispanic

^d ^Excludes sexual assault cases

**Fig. 4:**
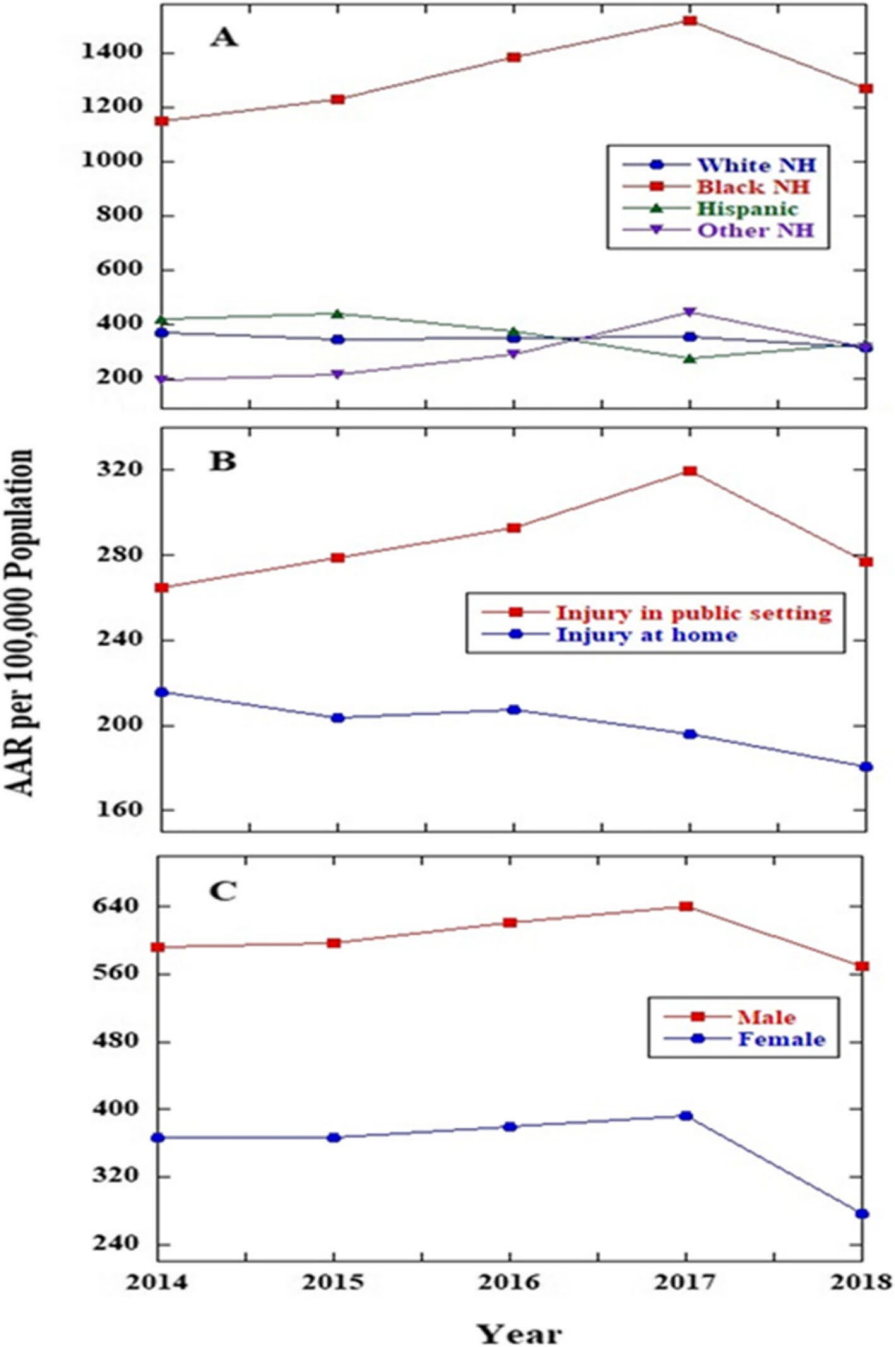
Assessing disparities using imputed NEISS-AIP companion data: trends in age-adjusted^a^ rates per 100,000 population of nonfatal assault injuries treated in emergency departments by (**A**) race/ethnicity, (**B**) location of injury, and (**C**) Sex from 2014 to 2018, United States. ^a^Age-adjusted to the 2000 U.S. standard population. Abbreviations: NEISS-AIP, National Electronic Injury Surveillance System-All Injury Program; AAR, Age-adjusted rate; NH, non-Hispanic

Figure 4 shows the trend for age-adjusted rates per 100,000 persons of nonfatal assault injuries treated in emergency departments by different groups from 2014 to 2018. The significantly higher nonfatal assault injury rates were found for Black NH persons compared with other race/ethnicity categories, regardless of year. In addition, the age-adjusted rates (AAR) for Black NH persons increase significantly from 2014 through 2017 (1147.9 in 2014 vs. 1519.7 in 2017), followed by a significant decline in 2018 (1271.7). Similar trends are also observed for nonfatal assault injuries occurring in public settings and for males. For nonfatal assault injuries occurring in public settings, AAR increased from 2014 through 2017 (264.5 in 2014 vs. 319.9 in 2017) and significantly declined in 2018 (276.8). For males, AAR increased from 2014 to 2017 (592.6 in 2014 vs. 640.1 in 2017) and then declined significantly in 2018 (570.2).

## Discussion

Eliminating health disparities is a central focus of Healthy People 2030 [38]. Injuries are a major public health concern, which cause over 200,000 deaths, and 30 million individuals are treated for injuries in hospitals and emergency departments each year [39]. Significant inequities exist in injury prevention and control in the US as demonstrated by health disparities across age, race, ethnicity, region, sex, etc. Preventing injuries in high-risk groups can have the biggest influence on achieving health equity in injury. To reduce and ultimately eliminate these disparities, the first step is understanding what these disparities are and who is affected by them. Investigating disparities requires an accurate identification and categorization of individuals into different subgroups [3]. Not having key indicators due to missingness, however, is a limitation on the use of NEISS-AIP in investigating disparities in injury. When data are not available, imputation is a method to attribute missing characteristics to specific observations in a data set. To make NEISS-AIP a more useful and reliable data source for the study of health disparities, it is essential to generate imputed companion data that will allow public users to perform analysis on complete datasets.

Conducting impactful research on injury disparities requires availability of comprehensive data. Statistical methods for addressing missing values have been actively pursued, including maximum likelihood estimation, Bayesian estimation, and MI. MI is the only technique that is computationally straightforward, versatile, relatively easy to apply, and increasingly available in standard statistical software. MI has arguably been the most popular method for handling missing data in practice [28, 40].

To identify the most appropriate MI model for handling missing data in NEISS-AIP, a comprehensive simulation study built on real non-missing data from NEISS-AIP 2018 was conducted. The FCS model performed better than the JM model for reporting overall racial/ethnic distributions that were closest to that of the standard control and had higher average correct prediction rates. We also developed a new *BSS* method to assess the accuracy of probabilistic predictions by different approaches for handling missing data. This intuitive *BSS* comparison clearly showed that FCS MI provided the most accurate imputed data for all missing categorical variables. This is the first application of the developed *BSS* method for assessing imputation performance quantitatively.

Because ignoring the missing race/ethnicity data influences the identification and magnitude of disparities, we further assessed bias by estimating crude rates of nonfatal assault injuries for White NH persons under different approaches for addressing high proportion missingness of race/ethnicity. Our simulation study provides strong evidence that FCS MI yields estimates that are unbiased and provide appropriate coverage. Unlike JM which assumes joint multivariate normality for all variables, FCS specifies the multivariate imputation model on a variable-by- variable basis by a set of conditional densities, one for each incomplete variable. FCS MI permits great flexibility because an appropriate imputation model can be selected for each missing variable [29].

Nonfatal assault injury is an important public health concern, imposing significant health care costs and productivity losses for millions of people each year [3, 13]. However, research on disparities in nonfatal assault injury has not been well characterized, limiting understanding of gaps in research and development of successful interventions. Much of the research on injury disparities in the United States focuses on the differences in injury rates [41]. In this study, we assessed whether disparities exist for age-adjusted average annual rates of nonfatal assault injury for ED visits by race/ethnicity, location of injury, and sex. Results showed that Black NH persons had significantly higher injury rate than other race/ethnicity categories (3.8 times higher than White NH persons, 3.6 times higher than Hispanic persons, and 4.5 times higher than Other NH persons) for the study period (2014–2018). Black NHs persons were at disproportionately high risk for nonfatal assault injuries compared with other race/ethnicity groups. These results underscore the importance of understanding and addressing the underlying inequities, such as limited educational, housing, and occupational opportunities, concentrated poverty, systemic racism, and other aspects of social and economic disadvantage, that contribute to risk for violence [42, 43]. Injuries occurring in public settings had significantly higher average annual rate than injuries occurring at home (1.4 times higher). Males had significantly higher average annual rate than females (1.6 times higher). The trends from 2014 to 2018 in age-adjusted nonfatal assault injury rates by different subgroups were also investigated. Similar trends were observed for Black NH persons, injuries occurring in public settings, and males: AARs of nonfatal assault injury increased significantly from 2014 through 2017, then declined significantly in 2018.

## Limitations

Study limitations exist. First, aggregated racial/ethnic groups were recorded in our analysis. Hispanic origin and race were combined into a single-item format to make them compatible with available annual bridged-race population estimates used as denominators for the injury rates. However, collection and reporting of ethnic and racial identity as two separate constructs is usually preferred, further disaggregating race/ethnicity may assist in better understanding disparities and in development of culturally responsive interventions [4]. Second, the statistical literature has defined three types of missingness mechanisms: missing completely at random (MCAR), missing at random (MAR), and missing not at random (MNAR). MI methods generally assume that the data is at least MAR, and therefore remains valid if observations are MCAR. The MAR assumption is generally considered to be realistic for well-conducted surveys and has been recommended for practical applications [27]. The assumption of MAR becomes more reasonable as more predictors are included in the imputation model [44]. As the NEISS-AIP contain high-quality data with a large amount of predictive information, the MAR assumption can be justified. However, it is impossible to determine whether data are MNAR solely based on observed data. The NEISS-AIP are de‐identified to prevent tracking patients for follow‐up or prior information, so the MNAR assumption cannot be tested. Bias caused by data that are MNAR can be addressed only by sensitivity analyses examining the effect of different assumptions about the missing data mechanism, which is outside the scope of this study. Third, the estimated non-fatal assault injury rates in this study are underestimates of the actual prevalence because data are limited to patients treated in hospital EDs and do not include those who had injuries treated in other health care settings (e.g., physician’s office or urgent care center) or those for whom no treatment was needed or sought. Accordingly, our findings may understate disparities in nonfatal assault injury risk.

## Conclusions

This study is the first to look at health disparities in nonfatal assault injuries using multiply imputed companion data. The developed methods and imputed data sets can also be applied to addressing disparities in other types of injuries. Groups at higher risk of an injury outcome could be identified for local prevention efforts. Health disparities are often viewed through the lens of race and ethnicity, but they occur across a broad range of dimensions. It is necessary to address the underlying social and economic inequities that drive disparities. Communities can make use of the best available evidence to prevent multiple forms of injury [45].

In summary, assessing disparities in injury is crucial for injury prevention and for evaluating injury prevention strategies. We assessed health disparities in nonfatal assault injury by generating multiply imputed companion data. Beyond the methodological insights, this study will also help to advance health disparities research by enhancing efforts to quantify, monitor, and develop solutions for assessing disparities in injury.

### Supplementary Information

**Additional file 1. **The Supplementary Material for this article can be found.

## Data Availability

The supporting data can be made available upon reasonable request.

## Appendix

### New BSS method for evaluating imputation performance

The Brier Score (*BS*), which the *BSS* is developed from, is a measure of the mean-square error of prediction [33]. In the observed events *N*, let *X* be a *K*-level (*K* > 1) categorical variable with *M* missing observations *x*_i_ (*i* = 1,….*M*). Denote *p*_ij_ the predicted probability of a specific level *j* (*j* = 1,….* K*) of *X*.

For MI with 20 iterations, $p\mathrm{ij }= \sum_{l=1}^{20}(x\mathrm{il} =j)/20$, where *x*_il_ is the imputed value of *x*_i_ from the *l*th iteration, *l* = 1,…20. The *BS* for MI is given as:

1 $$\begin{array}{cc}BS=\frac1N\sum_{i=1}^M\sum_{j=1}^K{(I\mathrm{ij}-p\mathrm{ij})}^2,&(0\leq BS\leq1)\end{array}$$

where *I*_ij_ = 1(0), if the true value of *x*_i_ is (not)* j*. *BS* has a range of 0 to 1, a smaller *BS* (closer to zero) implies a more precise imputation.

For CCA, denote *p*_i_, the predicted probability of *X*. We assume that all the predictions of missing values in the categorical variable *X* are wrong $(\mathrm{xi}\ne \mathrm{ j})$, which is the worst prediction case. The *BS*_*ref*_ for CCA is given as:

2 $$\begin{array}{cc}BSref=\frac1N\sum\nolimits_{i=1}^M{(Ii-pi)}^2&(0\leq BSref\leq1)\end{array}$$

where *I*_i_ = 1(0), if the true value of *x*_i_ is (not)* j*.

A noted shortcoming of *BS* is that the score value is often hard to interpret, despite it being a widely used scalar summary of accuracy for probability prediction. Consequently, the *BS* is frequently converted to a skill score, normalizing the score by that of a reference prediction strategy. *BSS* indicates the degree of skill improvement. It is designed to range from 1.0 for a perfect prediction strategy, through 0.0 for one that offers no improvement over the reference strategy, to negative values for strategies that are worse than the reference strategy [34, 35]:

3 $$\begin{array}{cc}BSS=1- \frac{BS}{BSref} ,& (-\infty \le BSS\le 1)\end{array}$$

Negative skill scores often need to be interpreted with caution as they may hide useful information contained in the prediction. However, negative skill scores can be avoided by selecting the appropriate reference strategy. In this study, we chose CCA as the reference strategy (*BS*_*ref*_) to avoid the negative skill scores for the two MI methods. Thus, *BSS* ranges from 0 to 1, where 0 means no accuracy improvement and 1 means a perfect accuracy improvement. As *BSS* approaches 1, this implies more accurate predictions for discrete outcomes in missing variables.
