# Supplementary material for: Addressing health disparities using multiply imputed injury surveillance data
Source: Int J Equity Health. 2023 Jul 3;22:126. doi: 10.1186/s12939-023-01940-4 (PMC10316636; doi:10.1186/s12939-023-01940-4)
Supplement: Supplementary file 1 — Additional file 1. The Supplementary Material for this article can be found. [file 12939_2023_1940_MOESM1_ESM.docx]

**Addressing health disparities using multiply imputed injury surveillance data**

**Supporting Information:** This supplement is intended to provide additional information on the details of the study.

**Table of Contents**

1. **Supplementary Text A:** The general procedure for multiple imputation. -------------- P. 2
2. **Table S1.** Summary of All Covariates used in the Multiple Imputation Model for imputing NEISS-AIP Data—United States, 2014–2018. ------------------------------------------ P. 3
3. **Table S2.** Missing data pattern analysis for NEISS-AIP 2018 data. -------------------**--** P. 4-5
4. **Figure S1.** Comparison of overall distributions of race/ethnicity before imputation (simulation data) and after imputations (JM imputation data and FCS imputation data) with the standard control dataa in simulation study. ------------------------------------------ P. 6
5. **Supplementary Text B:** Example SAS code for proc MI using FCS statement. --------- P. 7

**Supplementary Material A:** The general procedure for multiple imputation.

MI follows three distinct phases: 1) fill in the missing data *m* times to generate *m* complete datasets in which missing values are replaced with a set of plausible values based on their posterior predictive distribution given the observed data and the pattern of missing data; 2) analyze each complete dataset separately to obtain desired parameter estimates and SEs; and 3) combine results of the *m* analyses into a single set of statistics by computing the mean of the *m* estimates and the variance estimate that includes both within and between imputation variations to appropriately reflect the uncertainty associated with the imputed values. The imputation number *m* is determined by the missing proportion (*p*) and the desired relative efficiency (*RE*). *RE* is an indicator of how well the true population parameters are estimated, which is a function of *m* and *p* [25,26]:

*RE* ≈ *m*/(*m*+*p*). (1)

To achieve a greater than 95% *RE* in our study, we chose *m* as 20, resulting in 20 complete imputed datasets.

The estimates from the imputed datasets were combined to generate a single set of estimates using the SAS PROC MIANALYZE procedure. The overall estimate was the average of the estimates of the 20 complete imputed data sets. The variance of that overall estimate was a function of variance within each imputed dataset and of the variance across datasets [31]:

*Var*_total_ (*θ*) = ∑ *Var* _within_ (*θ*) + (1 + $\frac{1}{m}$ ) *Var* _between_ (*θ*) (2)

**Table S1.** Summary of All Covariates used in the Multiple Imputation Model for imputing NEISS-AIP^a^ Data—United States, 2014–2018.

| **Variable Name** | **Variable Type** | **Description** |
| --- | --- | --- |
| AGE | Numeric | Patient age in year |
| SEX | Binary | Sex of patient |
| RACE | Categorical | Patient race/ethnicity |
| DIAG | Categorical | Diagnosis |
| PCAUSE | Categorical | Precipitating cause of injury |
| BDYPT | Categorical | Primary body part affected |
| DISP | Categorical | Disposition of case |
| LOC | Categorical | Location where injured |
| TYPE | Categorical | Injury work related |
| INTENT | Categorical | Intent of injury |
| FMV | Categorical | Fire involvement |
| PSU | Numeric | Primary sampling unit |
| STRATUM | Categorical | Stratum based on size |
| SPORTS | Categorical | Sports and recreation related |
| PROD | Numeric | Product code |
| HOSP | Categorical | Hospital |
| TRDATE | Numeric | Date of treatment |
| INJURY | Categorical | Injury as defined by CDC |
| VIOLEN | Categorical | Violence injury from intention |
| YEAR | Numeric | Survey year |

^a^  NEISS-AIP: National Electronic Injury Surveillance System-All Injury Program.

**Table S2.** Missing data pattern analysis for NEISS-AIP 2018 data.^a^

| **Missing Data Pattern**  (X observed; • missing) | | | | | | | | | | |
| --- | --- | --- | --- | --- | --- | --- | --- | --- | --- | --- |
| **Group^b^** | **AGE** | **SEX** | **RACE** | **CAUSE** | **BDYPT** | **DISP** | **LOC** | **TYPE** | **Freq** | **Percent^c^**  **(%)** |
| 1 | X | X | X | X | X | X | X | X | 256566 | 44.17 |
| 2 | X | X | X | X | X | X | . | X | 113888 | 19.6 |
| 3 | X | X | . | X | X | X | X | X | 112454 | 19.36 |
| 4 | X | X | . | X | X | X | . | X | 67044 | 11.54 |
| 5 | X | X | X | . | X | X | X | X | 6563 | 1.13 |
| 6 | X | X | X | X | . | X | X | X | 5441 | 0.94 |
| 7 | X | X | X | . | X | X | . | X | 4887 | 0.84 |
| 8 | X | X | . | X | . | X | X | X | 2600 | 0.45 |
| 9 | X | X | X | X | X | X | . | . | 2395 | 0.41 |
| 10 | X | X | . | . | X | X | . | X | 2081 | 0.36 |
| 11 | X | X | . | . | X | X | X | X | 1988 | 0.34 |
| 12 | X | X | . | X | X | X | . | . | 1540 | 0.27 |
| 13 | X | X | X | X | . | X | . | X | 1220 | 0.21 |
| 14 | X | X | X | X | X | X | X | . | 918 | 0.16 |
| 15 | X | X | . | X | . | X | . | X | 379 | 0.07 |
| 16 | X | X | X | . | X | X | . | . | 301 | 0.05 |
| 17 | X | X | . | X | X | X | X | . | 145 | 0.02 |
| 18 | X | X | X | X | . | X | . | . | 31 | 0.01 |
| 19 | X | X | X | . | . | X | X | X | 74 | 0.01 |
| 20 | X | X | X | . | . | X | . | X | 37 | 0.01 |
| 21 | X | X | . | . | X | X | . | . | 42 | 0.01 |
| 22 | . | X | X | X | X | X | X | X | 56 | 0.01 |
| 23 | . | X | X | X | X | X | . | X | 39 | 0.01 |
| 24 | . | X | . | X | X | X | X | X | 54 | 0.01 |
| 25 | X | X | X | X | X | . | X | X | 3 | 0 |
| 26 | X | X | X | X | X | . | . | X | 3 | 0 |
| 27 | X | X | X | X | . | X | X | . | 22 | 0 |
| 28 | X | X | X | . | X | X | X | . | 28 | 0 |
| 29 | X | X | X | . | . | X | . | . | 1 | 0 |
| 30 | X | X | . | X | X | . | X | X | 3 | 0 |
| 31 | X | X | . | X | X | . | . | X | 4 | 0 |
| 32 | X | X | . | X | . | X | X | . | 5 | 0 |
| 33 | X | X | . | X | . | X | . | . | 12 | 0 |
| 34 | X | X | . | X | . | . | . | X | 1 | 0 |
| 35 | X | X | . | . | X | X | X | . | 5 | 0 |
| 36 | X | X | . | . | X | . | . | X | 1 | 0 |
| 37 | X | X | . | . | . | X | X | X | 25 | 0 |
| 38 | X | X | . | . | . | X | . | X | 9 | 0 |
| 39 | X | X | . | . | . | X | . | . | 2 | 0 |
| 40 | X | . | X | X | X | X | X | X | 3 | 0 |
| 41 | X | . | X | X | X | X | . | X | 1 | 0 |
| 42 | X | . | . | X | X | X | X | X | 2 | 0 |
| 43 | X | . | . | X | X | X | . | X | 2 | 0 |
| 44 | X | . | . | X | . | X | X | X | 1 | 0 |
| 45 | . | X | X | X | X | X | X | . | 1 | 0 |
| 46 | . | X | X | X | X | X | . | . | 2 | 0 |
| 47 | . | X | X | X | . | X | . | X | 2 | 0 |
| 48 | . | X | X | . | X | X | X | X | 2 | 0 |
| 49 | . | X | . | X | X | X | . | X | 26 | 0 |
| 50 | . | X | . | . | X | X | X | X | 1 | 0 |
| 51 | . | X | . | . | X | X | . | X | 1 | 0 |
| 52 | . | . | X | X | X | X | X | X | 3 | 0 |
| 53 | . | . | X | X | X | X | . | X | 1 | 0 |
| 54 | . | . | . | X | X | X | X | X | 4 | 0 |
| 55 | . | . | . | X | X | X | . | X | 6 | 0 |

^a^ Fifty-four total patterns with at least one missing variable were obtained from the National Electronic Injury Surveillance System-All Injury Program (NEISS-AIP) 2018 data.

^b^ LOC: location where the injury occurred.

RACE: race and ethnicity.

CAUSE: external cause of injury.

BDYPT: primary body part affected.

TYPE: work-relatedness.

AGE: age in years.

DISP: disposition at emergency department discharge.

SEX: gender.

^c^ Percentages were calculated among total observations of NEISS-AIP 2018 data.

**Figure S1.** Comparison of overall distributions of race/ethnicity before imputation (simulation data^a^) and after imputations (JM imputation data and FCS imputation data) with the standard control data in simulation study.

**
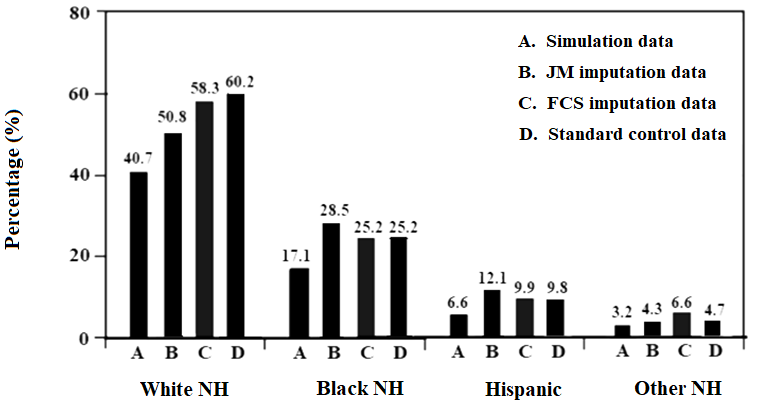
**

^a^  Simulation data was developed by imposing the missing data patterns on the subset of fully observed data in the National Electronic Injury Surveillance System-All Injury Program (NEISS-AIP), 2018. Non-missing data (the fully observed subset data) was used as the standard control. A represents the complete case analysis (CCA) of the simulation data.

**Abbreviations:** JM, joint modeling; FCS, fully conditional specification; NH, non-Hispanic.

**Supplementary Text B:** Example SAS code for proc MI using FCS statement.

PROC MI DATA=DATAIN MINIMUM=MINVALUE MAXIMUM=MAXVALUE ROUND=1

NIMPUTE=m OUT=DATAOUT;

CLASS Y1 Y2 Y3 Y4;

FCS PLOTS=TRACE

REG(X1/details)

LOGISTIC(Y1/details);

VAR X1-X3 Y1-Y4;

RUN;

Note:

**MINIMUM** or **MAXIMUM** is the command for specifying the bound value to be imputed for the variables, where you can specify a number for all the variables being imputed or only for certain ones.

**ROUND** is to round the imputed value.

**NIMPUTE** is to select the number of imputations (m).

**X1-X3** and **Y1-Y4** represent the continuous and categorical variables correspondingly.

The **FCS** approach indicates what specific model to be used given the type of variable imputed.

**PLOTS=TRACE** will display trace plots for worst linear function, variable means, variable variances, and covariances of variables.

**VAR** statement shows the desired variables used in the imputation model.
